# Artesunate Enhances the Cytotoxicity of 5-Aminolevulinic Acid-Based Sonodynamic Therapy against Mouse Mammary Tumor Cells In Vitro

**DOI:** 10.3390/molecules22040533

**Published:** 2017-03-27

**Authors:** Tomohiro Osaki, Yoshihiro Uto, Masahiro Ishizuka, Tohru Tanaka, Nobuyasu Yamanaka, Tsukasa Kurahashi, Kazuo Azuma, Yusuke Murahata, Takeshi Tsuka, Norihiko Itoh, Tomohiro Imagawa, Yoshiharu Okamoto

**Affiliations:** 1 Joint Department of Veterinary Clinical Medicine, Faculty of Agriculture, Tottori University, Tottori 680-8553, Japan; kazu-azuma@muses.tottori-u.ac.jp (K.A.); ymurahata@muses.tottori-u.ac.jp (Y.M.); tsuka@muses.tottori-u.ac.jp (T.T.); taromobile@me.com (N.I.); imagawat@muses.tottori-u.ac.jp (T.I.); yokamoto@muses.tottori-u.ac.jp (Y.O.); 2 Faculty of Bioscience and Bioindustry, Tokushima University, Tokushima 770-8506, Japan; uto.yoshihiro@tokushima-u.ac.jp; 3 SBI Pharmaceuticals Co., Ltd., Tokyo 106-6020, Japan; mishizuk@sbigroup.co.jp (M.I.); tortanaka@sbigroup.co.jp (T.T.); 4 ITO Co., Ltd., Tokyo 176-0014, Japan; yamanaka@itolator.co.jp (N.Y.); kurahashi@itolator.co.jp (T.K.)

**Keywords:** 5-aminolevulinic acid, artesunate, breast cancer, sonodynamic therapy, ultrasound

## Abstract

Sonodynamic therapy (SDT) kills tumor cells through the synergistic effects of ultrasound (US) and a sonosensitizer agent. 5-Aminolevulinic acid (5-ALA) has been used as a sonodynamic sensitizer for cancer treatment. However, studies have shown that 5-ALA-based SDT has limited efficacy against malignant tumors. In this study, we examined whether artesunate (ART) could enhance the cytotoxicity of 5-ALA-based SDT against mouse mammary tumor (EMT-6) cells in vitro. In the ART, ART + US, ART + 5-ALA, and ART + 5-ALA + US groups, the cell survival rate correlated with ART concentration, and decreased with increasing concentrations of ART. Morphologically, many apoptotic and necrotic cells were observed in the ART + 5-ALA + US group. The percentage of reactive oxygen species-positive cells in the ART + 5-ALA + US group was also significantly higher than that in the 5-ALA group (*p* = 0.0228), and the cell death induced by ART + 5-ALA + US could be inhibited by the antioxidant *N*-acetylcysteine. These results show that ART offers great potential in enhancing the efficacy of 5-ALA-based SDT for the treatment of cancer. However, these results are only based on in vitro studies, and further in vivo studies are required.

## 1. Introduction

Breast cancer is now the most frequently diagnosed cancer and one of the most deadly diseases for women [1]. Conventionally, treatments for breast cancer include surgery, radiation therapy, chemotherapy, and hormone therapy. Sonodynamic therapy (SDT) is a relatively new approach in treating cancer. SDT utilizes the synergistic effects of ultrasound (US) and a sonosensitizer to kill tumor cells. Photosensitizers such as porphyrins, chlorins, and phthalocyanines, which are typically used in clinical photodynamic therapy, have been extensively studied for use in SDT [2]. An example of one such sonosensitizer is protoporphyrin IX (PpIX), which is derived from the pro-drug 5-aminolevulinic acid (5-ALA). Multiple studies have demonstrated that 5-ALA-induced PpIX can generate reactive oxygen species (ROS) when exposed to US, suggesting that 5-ALA-based SDT may be a potential therapeutic modality for the management of malignant tumors [3,4]. In support of this hypothesis, our previous report suggested that 5-ALA-based SDT induces an antitumor effect in mouse mammary tumor cells through oxidation of the mitochondrial membrane via ROS production [5]. Although 5-ALA-based SDT is effective against malignant tumors, it may be insufficient to cure cancer. Therefore, improving the efficacy of 5-ALA-based SDT is of vital importance.

We previously reported that the efficacy of AlPcS2a- or 5-ALA-based SDT can be enhanced by bleomycin (BLM) [6,7], an effective antineoplastic drug that binds to iron and oxygen to produce ROS [8]. This suggests that combining SDT with administration of antineoplastic drugs is a promising method for improving the efficacy of 5-ALA-based SDT. However, the repeated systemic administration of BLM can result in lung inflammation, which can progress to fibrosis [9,10]. Therefore, an alternative antineoplastic drug with lower toxicity is highly desirable.

Artemisinin, a chemical compound derived from *Artemisia annua*, has been established as a successful treatment for malaria, and is known to exert antineoplastic effects. The anti-malarial and anti-cancer effects of artemisinin are attributable to its endoperoxide moiety, with the endoperoxide bond most likely activated by reduced heme or ferrous iron, leading to cytotoxic carbon-centered radicals, which are strong alkylating agents [11,12]. Various derivatives of artemisinin, including artesunate (ART), artemether, dihydroartemisinin, and arteether, have been identified [13,14]. ART is a semi-synthetic derivative of artemisinin, and is a more effective antimalarial agent [15]. It also exhibits anti-cancer activity against a variety of cancer cells, and has been reported to rapidly convert to ROS inside cells, thereby disrupting cellular functions [16]. ART is well-tolerated by patients, with little toxicity and no obvious side effects [17]. Therefore, ART is a promising candidate in the search for a low-toxicity compound that would increase the efficacy of 5-ALA-based SDT.

Based on these findings, we examined whether ART could enhance the cytotoxicity of 5-ALA-based SDT against mouse mammary tumors as a human cancer model in vitro.

## 2. Results

### 2.1. Cytotoxicity Analysis of ART

**Figure 1:**
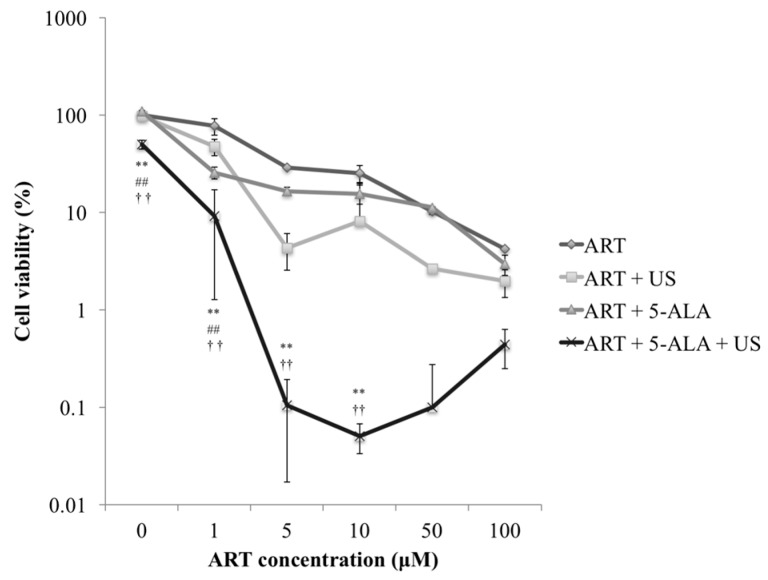
Cytotoxicity analysis of the artesunate (ART) dose. EMT-6 cells were incubated with 0, 1, 5, 10, 50, or 100 μM ART for 24 h. In the ART + 5-aminolevulinic acid (5-ALA) and ART + 5-ALA + ultrasound (US) groups, the cells were further incubated with 1 mM 5-ALA for 4 h. After rinsing with fresh medium, the cells were exposed to US. The tumor cells were sonicated for 60 s at a frequency of 3 MHz and a power intensity of 3 W/cm^2^, and a 20% duty cycle was used. Subsequently, the cells were re-incubated at 37 °C for 24 h in the dark. ** *p* < 0.01: ART + 5-ALA + US group vs. ART, ## *p* < 0.01: ART + 5-ALA + US group vs. ART + US, †† *p* < 0.01: ART + 5-ALA + US group vs. ART + 5-ALA. Results are presented as the mean ± standard deviation.

Figure 1 shows the results of the cytotoxicity analysis with various concentrations of ART. The most notable decrease in cell survival was observed when ART concentrations were between 0 and 5 μM. The ART + 5-ALA + US treatment induced 99% cell death when ART concentration exceeded 5 μM.

At 0 μM ART, the cell viability in the ART + 5-ALA + US group decreased significantly compared with viability in the ART, ART + US, and ART + 5-ALA groups (*p* < 0.0001 for all comparisons). At 1 μM ART, the cell viability in the ART + 5-ALA + US group decreased significantly compared with viability in the ART, ART + US, and ART + 5-ALA groups (*p* < 0.0001, *p* < 0.0001, and *p* = 0.0009, respectively). Furthermore, the cell viability in the ART + 5-ALA group decreased significantly compared with viability in the ART and ART + US groups (*p* < 0.0001 for both comparisons); cell viability in the ART + US group decreased significantly compared with viability in the ART group (*p* < 0.0001).

At 5 μM ART, the cell viability in the ART + 5-ALA + US group decreased significantly compared with viability in the ART and ART + 5-ALA groups (*p* < 0.0001 and *p* = 0.0039, respectively). Moreover, the cell viability in the ART + US group decreased significantly compared with viability in the ART group (*p* < 0.0001), and the cell viability in the ART + US group decreased significantly compared with viability in the ART + 5-ALA group (*p* = 0.0343). At 10 μM ART, the cell viability in the ART + 5-ALA + US group decreased significantly compared with viability in the ART and ART + 5-ALA groups (*p* < 0.0001 and *p* = 0.0069, respectively). Furthermore, the cell viability in the ART + US group decreased significantly compared with viability in the ART group (*p* = 0.0027).

### 2.2. In Vitro Evaluation of 5-ALA-Based SDT with ART

**Figure 2:**
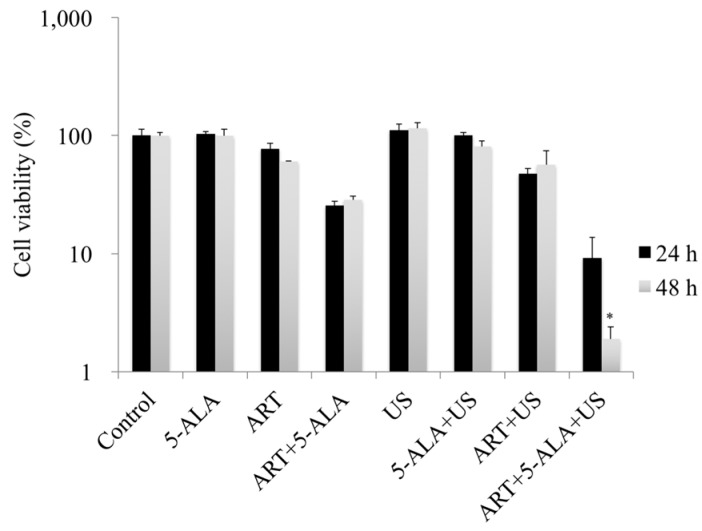
In vitro assessment of 5-aminolevulinic acid (5-ALA)-based sonodynamic therapy (SDT) with artesunate (ART). EMT-6 cells were incubated with 1 μM ART or 1 mM 5-ALA for 24 or 48 h, and then with 1 mM 5-ALA for a further 4 h. After rinsing with fresh medium, the cells were exposed to ultrasound (US). Cells were sonicated for 60 s at a frequency of 3 MHz and a power intensity of 3 W/cm^2^, and a 20% duty cycle was used. Subsequently, the cells were re-incubated at 37 °C for 24 h in the dark. Following incubation, the cells were harvested and prepared for cell counting. * *p* < 0.05: ART + 5-ALA + US group vs. US. Results are presented as the mean ± standard deviation.

After incubation with ART for 24 h, the cell viabilities in the control, 5-ALA, ART, and ART + 5-ALA groups were 100%, 103.2%, 77.0%, and 25.6%, respectively. The cell viabilities in the US, 5-ALA + US, ART + US, and ART + 5-ALA + US groups were 111.3%, 100.5%, 47.3%, and 9.1%, respectively (Figure 2). After incubation with ART for 48 h, the cell viabilities in the control, 5-ALA, ART, and ART + 5-ALA groups were 100%, 100%, 60.3%, and 28.4%, respectively. The cell viabilities in the US, 5-ALA + US, ART + US, and ART + 5-ALA + US groups were 115.1%, 80.9%, 56.6%, and 1.9%, respectively (Figure 2). The cell viability in the ART + 5-ALA + US group decreased significantly compared with viability in the US group (*p* = 0.0228).

### 2.3. Morphological Changes in EMT-6 Cells

**Figure 3:**
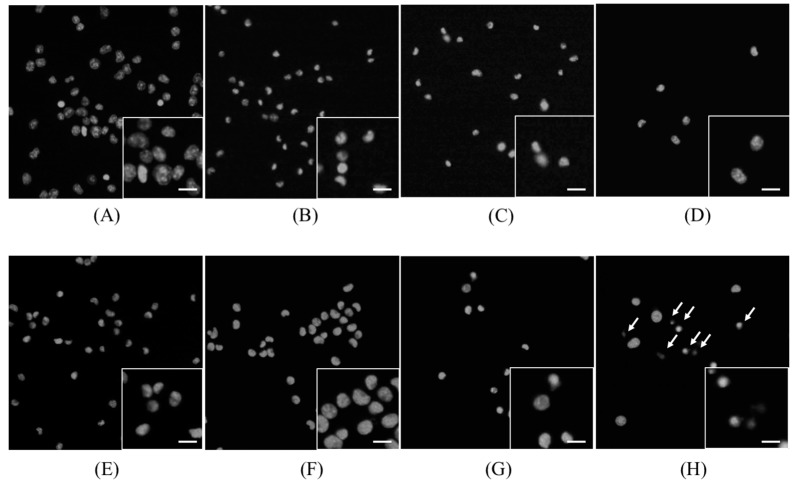
Hoechst 33342 dye staining images. (**A**) Control group; (**B**) 5-aminolevulinic acid (5-ALA) group; (**C**) artesunate (ART) group; (**D**) ART + 5-ALA group; (**E**) ultrasound (US) group; (**F**) sonodynamic therapy (SDT) group, (**G**) ART + US group, (**H**) ART + 5-ALA + US group. In the ART + 5-ALA + US group, condensation of the nuclear chromatin was also observed (indicated by arrows). Scale bar in magnified inserts: 10 μm.

Morphological changes in the cells were observed 4 h after sonication. Figure 3 shows the images obtained after staining with Hoechst 33342 dye. In the ART + 5-ALA, ART + US, and ART + 5-ALA + US groups, a decrease in cell number was observed. In the ART + 5-ALA + US group, condensation of the nuclear chromatin was also observed.

**Figure 4:**
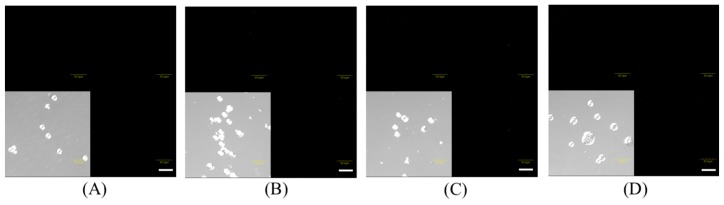

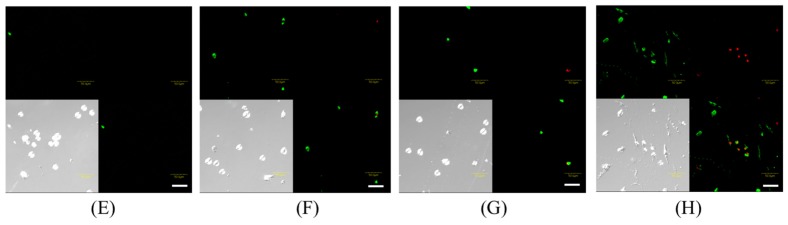
Annexin V and EthD-III double staining images. (**A**) Control group; (**B**) 5-aminolevulinic acid (5-ALA) group; (**C**) artesunate (ART) group; (**D**) ART + 5-ALA group; (**E**) ultrasound (US) group; (**F**) sonodynamic therapy (SDT) group; (**G**) ART + US group; (**H**) ART + 5-ALA + US group. In the US, ALA + US, and ART + US groups, a few early apoptotic cells were observed. In the ART + 5-ALA + US group, many late apoptotic and necrotic cells were observed. Red: Stained with ethidium homodimer III (EthD-III). Green: stained with Annexin V-fluorescein isothiocyanate (FITC). Mixture: Stained with both EthD-III and Annexin V-FITC. Scale bar in magnified inserts: 50 μm.

Figure 4 shows the images obtained after double staining with Annexin V and ethidium homodimer III (EthD-III). A few apoptotic cells were observed in the US, ALA + US, and ART + US groups, and many apoptotic and necrotic cells were observed in the ART + 5-ALA + US group. In contrast, no morphological signs of apoptosis were observed in the control, 5-ALA, ART, and ART + 5-ALA groups.

### 2.4. ROS Assay

**Figure 5:**
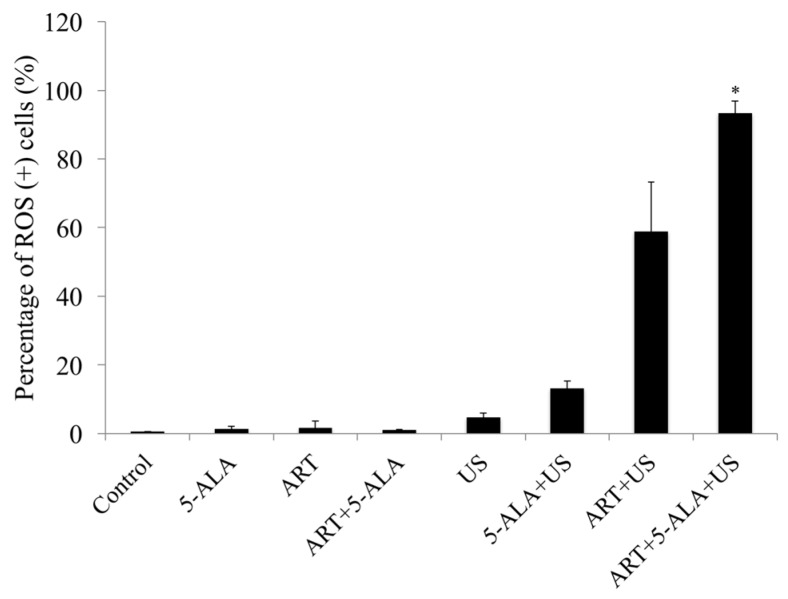
Reactive oxygen species (ROS) assay. The percentage of ROS-positive cells in the artesunate (ART) + 5-aminolevulinic acid (5-ALA) + ultrasound (US) group was significantly higher than that in the control group. * *p* < 0.05: ART + 5-ALA + US group vs. Control. Results are presented as the mean ± standard deviation.

The percentage of ROS-positive cells in the control, 5-ALA, ART, and ART + 5-ALA groups was 0.5, 1.4, 1.6, and 1.0, respectively. The percentage of ROS-positive cells in the US, ALA + US, ART + US, and ART + 5-ALA + US groups was 4.7, 13.1, 58.9, and 93.3, respectively. The percentage of ROS-positive cells in the ART + 5-ALA + US group was significantly higher than the percentage in the control group (*p* = 0.0228) (Figure 5).

### 2.5. ROS Mediate ART + 5-ALA + US-Induced Cell Death

**Figure 6:**
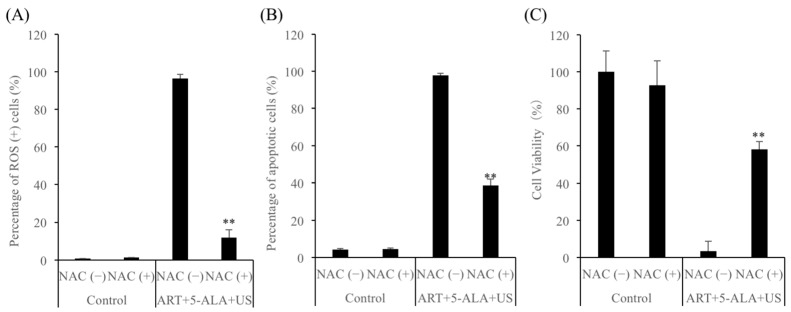
The cytotoxicity in the presence or absence of N-acetylcysteine (NAC). EMT-6 cells were incubated with 1 μM artesunate (ART) for 24 h, and then with 1 mM 5-aminolevulinic acid (5-ALA) for a further 4 h in the presence (+) or absence (−) of 15 mM NAC. After rinsing with fresh medium, the cells were exposed to ultrasound (US). Cells were sonicated for 60 s at a frequency of 3 MHz and a power intensity of 3 W/cm^2^, and a 20% duty cycle was used. Subsequently, the cells were re-incubated at 37 °C for 4 h in the dark. Following incubation, the cells were harvested and prepared for cell analysis. (**A**) The percentage of reactive oxygen species (ROS)-positive cells (%); (**B**) The percentage of apoptotic cells; (**C**) The cell viability (%). ** *p* < 0.01: ART + 5-ALA + US group vs. Control. Results are presented as the mean ± standard deviation.

To further investigate whether ART + 5-ALA + US-induced ROS are required for the induction of cell death, EMT-6 cells were treated with ART + 5-ALA + US in the presence (15 mM) or absence of the antioxidant N-acetylcysteine (NAC). The induction of ROS by ART + 5-ALA + US could be inhibited by NAC (Figure 6A). However, ART + 5-ALA + US-induced apoptosis and cell death could not be completely blocked by NAC (Figure 6B,C).

## 3. Discussion

SDT is a new and effective approach for the treatment of malignant tumors. Li et al. recently reported that pretreatment with 5-ALA (2 mM) could effectively enhance the toxicity of US sonication (sonication time, 7 min; intensity, 2 W/cm^2^; frequency, 1 MHz) in rat osteosarcoma (UMR-106) cells. The survival rate of UMR-106 cells after 5-ALA-based SDT was 64.4%, which was significantly lower than that of the control cells, although it was still not high enough to represent an effective treatment [18]. Song et al. similarly reported that pretreatment with 5-ALA (10 μg/mL) could effectively enhance the toxicity of US sonication (sonication time, 2 min; frequency, 1.05 MHz) in SAS (human tongue carcinoma) cells. However, the apoptosis rate in the 5-ALA-SDT group only increased by 20.91% compared with apoptosis in the group treated with US alone [4]. From these findings, it could be concluded that although 5-ALA-based SDT is more effective than US treatment alone, it only has limited efficacy against malignant tumors. Therefore, the effect of 5-ALA-based SDT needs to be improved further. In our previous in vitro study, BLM was found to enhance the efficacy of 5-ALA-based SDT [6]. However, treatment with BLM can lead to serious complications, such as pulmonary fibrosis and impaired lung function. Therefore, in this study, we examined whether ART, which has low toxicity and no obvious side effects, could take the place of BLM as an SDT-enhancing agent.

The active moiety of ART is an endoperoxide bridge that generates carbon-centered free radicals and oxidative stress upon cleavage [19]. It has been reported that the intracellular bioactivation of the endoperoxide to carbon-centered radicals is dependent on cellular heme, with the addition of PpIX, a precursor of heme that can increase heme synthesis to levels approximately 2.5 times greater than normal, significantly increasing sensitivity to ART-induced cytotoxicity [20]. In other words, 5-ALA induces PpIX, which results in increased ART-induced cytotoxicity due to enhanced endoperoxide bioactivation.

In this study, ART was shown to enhance the cytotoxicity of 5-ALA-based SDT against mammary tumors. In the ART, ART + US, and ART + 5-ALA + US groups, the cell survival rate correlated with ART concentration, with the survival rate decreasing as the concentration of ART increased. It has previously been reported that ART suppresses the growth of several types of tumor in a dose-dependent manner [21,22]. In this study, the percentage of ROS-positive cells in the ART + 5-ALA + US group increased compared with that in the ART + 5-ALA group. ART + 5-ALA + US also induced apoptotic and necrotic cell death. Efferth et al. reported that the antioxidant NAC can almost completely block ART-induced apoptosis [23]. The induction of ROS by ART + 5-ALA + US was also inhibited by NAC. The effect of the ROS scavenger NAC on 5′-Cl-induced apoptosis can be detected using a Muse^®^ Oxidative Stress Kit [24].

It has been reported that ART may have distinct mechanisms of cytotoxicity in different cancer cell lines. ART induces apoptosis in HOS (human osteosarcoma) cells [25], and oncosis-like Panc-1 cell death [26]. It has also been reported that artemisinin derivatives, such as ART and dihydroartemisinin (DHA), can enhance antineoplastic effects in different cancer cell lines. For example, ART can induce radiosensitivity in HeLa (human cervical cancer) cells, both in vitro and in vivo [27]. DHA also augments photodynamic therapy-induced growth inhibition and apoptosis in esophageal cancer cells [28], and ART can synergize with doxorubicin to enhance leukemic T cell apoptosis [23]. Therefore, a detailed investigation of the effects and mechanism of action of ART + 5-ALA + US in different cell lines is required.

## 4. Materials and Methods

### 4.1. Cell Line and Culture Conditions

EMT-6 mouse mammary tumor cells (supplied by Dr. Shin-ichiro Masunaga, Kyoto University, Kyoto, Japan) were maintained as an adherent monolayer culture, and incubated in 5% CO_2_ at 37 °C. The culture medium used consisted of RPMI 1640 medium (Invitrogen, Carlsbad, CA, USA) supplemented with 10% heat-inactivated fetal bovine serum (Nichirei Biosciences Inc., Tokyo, Japan) and PSN (5 mg/mL penicillin, 5 mg/mL streptomycin, and 10 mg/mL neomycin; Invitrogen).

For the experiments, the cells were harvested from near-confluent cultures by brief exposure to a solution containing 0.25% trypsin and 1 mmol/L EDTA·4Na with phenol red (Invitrogen). The trypsinization was then stopped using RPMI 1640 medium containing 10% fetal bovine serum, and the cells were centrifuged and re-suspended in RPMI 1640 medium. Trypan blue staining was used to assess cell viability.

### 4.2. Chemicals

5-ALA was donated by SBI Pharma (Tokyo, Japan). A stock solution of 100 mM 5-ALA in phosphate-buffered saline (PBS), which was stored at 4 °C when not in use, was used for the in vitro experiments. Artesunate (Tokyo Chemical Industry Co., Ltd., Tokyo, Japan) was prepared in PBS, and diluted to the indicated final concentrations in the cell cultures.

### 4.3. US Exposure

**Figure 7:**
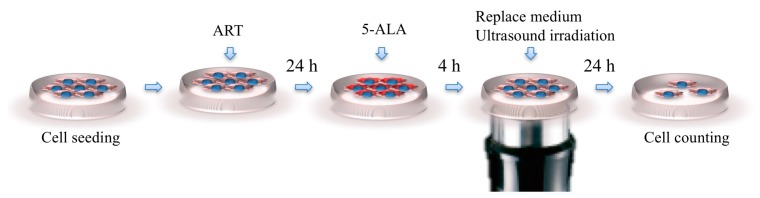
In vitro experimental scheme. The gap between the culture dish and the probe was filled with echo gel.

The cells were exposed to US (3 MHz, 3 W/cm^2^ output intensity, and 20% duty cycle for 60 s) using a US generator (UST-770, ITO Physiotherapy and Rehabilitation, Tokyo, Japan) with a single circular disk (35-mm diameter) as a US transducer. To expose the tumor cells to US in vitro, the 35-mm culture dishes were placed above a probe, and the gap between the culture dish and the probe was filled with echo gel (ITO Co., Ltd.; Figure 7).

### 4.4. Cytotoxicity Analysis of the ART Dose

We seeded 5 × 10^4^ EMT-6 cells into 35-mm Petri dishes (Nunc, Ltd., Roskilde, Denmark) containing 2 mL of culture medium. After 24 h of incubation, the dishes were divided into the following four groups: (1) ART group (treated with various concentrations of ART), (2) ART + US group (treated with various concentrations of ART, and then sonicated), (3) ART + 5-ALA group (treated with various concentrations of ART and 1 mM 5-ALA), and (4) ART + 5-ALA + US group (treated with various concentrations of ART and 1 mM 5-ALA, and then sonicated). The cells were protected from light throughout experiments involving 5-ALA.

The EMT-6 cells were incubated with 0, 1, 5, 10, 50, or 100 μM ART for 24 h. In the ART + 5-ALA and ART + 5-ALA + US groups, the cells were further incubated with 1 mM 5-ALA for 4 h. After rinsing with fresh medium, the cells were exposed to US (except for the control, ART, and ART + 5-ALA groups). The tumor cells were sonicated for 60 s at a frequency of 3 MHz and a power intensity of 3 W/cm^2^, and a 20% duty cycle was used. Subsequently, the cells were re-incubated at 37 °C for 24 h in the dark. Following incubation, the cells were harvested, and prepared for cell counting.

Cytotoxicity was determined using the Muse^TM^ Count and Viability Kit (EMD Millipore Co., Billerica, MA, USA) according to the manufacturer’s instructions. After the 24-h incubation post-sonication, the cells were trypsinized and incubated with the Muse^TM^ Count and Viability Reagent at room temperature for 5 min in the dark. Single-cell suspensions were then loaded onto the Muse Cell Analyzer (EMD Millipore Co.), and the cell viability was calculated using the following equation: (average of the test group/average of the control group) × 100.

### 4.5. Analysis of SDT with ART

We seeded 5 × 10^4^ EMT-6 cells into 35-mm Petri dishes containing 2 mL of culture medium. After 24 h of incubation, the dishes were divided into the following eight groups: (1) Control group (no treatment); (2) 5-ALA group (treated with 1 mM 5-ALA); (3) ART group (treated with 1 μM ART); (4) ART + 5-ALA group (treated with 1 μM ART and 1 mM 5-ALA); (5) US group (sonicated at a power intensity of 3 W/cm^2^); (6) SDT group (treated with 1 mM 5-ALA, and then sonicated); (7) ART + US group (treated with 1 μM ART, and then sonicated); and (8) ART + 5-ALA + US group (treated with 1 μM ART and 1 mM 5-ALA, and then sonicated). The cells were protected from light throughout experiments involving 5-ALA.

In the groups treated with a single compound (i.e., the 5-ALA, ART, 5-ALA + US, and ART + US groups), the EMT-6 cells were incubated with 1 μM ART or 1 mM 5-ALA for 24 or 48 h. In the groups treated with both compounds (i.e., the ART + 5-ALA and ART + 5-ALA + US groups), the EMT-6 cells were first incubated with 1 μM ART for 24 or 48 h, and then with 1 mM 5-ALA for 4 h. After rinsing with fresh medium, the cells were exposed to US (except for the control, 5-ALA, ART, and ART + 5-ALA groups). The tumor cells were sonicated for 60 s at a frequency of 3 MHz and a power intensity of 3 W/cm^2^, and a 20% duty cycle was used, as in our previous SDT experiments [6,7]. Subsequently, the cells were re-incubated at 37 °C for 24 h in the dark. Following incubation, the cells were harvested and prepared for cell counting. The cell viability was then determined as described in Section 2.4.

### 4.6. Morphological Changes in the EMT-6 Cells

We seeded 5 × 10^4^ EMT-6 cells into 35-mm Petri dishes containing 2 mL of culture medium. After 24 h of incubation, the dishes were divided into the following eight groups: (1) Control group (no treatment); (2) 5-ALA group (treated with 1 mM 5-ALA); (3) ART group (treated with 1 μM ART); (4) ART + 5-ALA group (treated with 1 μM ART and 1 mM 5-ALA); (5) US group (sonicated at a power intensity of 3 W/cm^2^); (6) SDT group (treated with 1 mM 5-ALA, and then sonicated); (7) ART + US group (treated with 1 μM ART, and then sonicated); and (8) ART + 5-ALA + US group (treated with 1 μM ART and 1 mM 5-ALA, and then sonicated). As in our previous SDT experiments, a 20% duty cycle was used. The cells were protected from light throughout experiments involving 5-ALA.

Apoptotic, necrotic, and healthy cells were then identified using a PromoKine Apoptotic/Necrotic/Healthy Cells Detection Kit, according to the manufacturer’s instructions (PromoKine, Heidelberg, Germany). The cells were stained with Hoechst 33342 dye 4 h after sonication. The nuclear morphology of the cells was then examined using an Olympus BX51 optical microscope (Olympus, Tokyo, Japan). To identify apoptotic and necrotic cells, the cells were stained with Annexin V-FITC and EthD-III according to the manufacturers’ instructions 4 h after sonication. The nuclear morphology of the cells was then examined using an Olympus Fluoview FV1000 confocal laser scanning microscope (Olympus). The cells were also analyzed by fluorescence microscopy using a fluorescein isothiocyanate (FITC) and Texas Red filter set.

### 4.7. ROS Assay

We seeded 5 × 10^4^ EMT-6 cells into 35-mm Petri dishes containing 2 mL of culture medium. After 24 h of incubation, the dishes were divided into the following eight groups: (1) Control group (no treatment); (2) 5-ALA group (treated with 1 mM 5-ALA); (3) ART group (treated with 1 μM ART); (4) ART + 5-ALA group (treated with 1 μM ART and 1 mM 5-ALA); (5) US group (sonicated at a power intensity of 3 W/cm^2^); (6) SDT group (treated with 1 mM 5-ALA, and then sonicated); (7) ART + US group (treated with 1 μM ART, and then sonicated); and (8) ART + 5-ALA + US group (treated with 1 μM ART and 1 mM 5-ALA, and then sonicated). A 20% duty cycle was used, and the cells were protected from light throughout experiments involving 5-ALA.

ROS generation was then assessed 4 h after sonication using a Muse^®^ Oxidative Stress Kit (EMD Millipore Co.) according to the manufacturer’s instructions. This kit determines the percentage of cells that are negative for ROS (healthy cells) and positive for ROS (cells containing ROS).

### 4.8. Effect of an Antioxidant on SDT-Induced Cell Death

EMT-6 cells were incubated with 1 μM ART for 24 h, and then with 1 mM 5-ALA for 4 h in the presence (15 mM) or absence of the antioxidant NAC. A 20% duty cycle was used, and the cells were protected from light throughout experiments involving 5-ALA.

ROS generation was assessed 4 h after sonication using a Muse^®^ Oxidative Stress Kit. The percentage of apoptotic cells was assessed 4 h after sonication using a Muse^®^ Annexin V and Dead Cell Assay Kit. Cytotoxicity was assessed 4 h after sonication using the Muse^TM^ Count and Viability Kit.

### 4.9. Statistical Analysis

Data were analyzed using Dunn’s multiple comparison test. A *p* value <0.05 was considered statistically significant. Statistical analyses were performed using GraphPad Prism version 6 (GraphPad Software Inc., La Jolla, CA, USA).

## 5. Conclusions

We found that ART enhanced 5-ALA-based SDT-induced EMT-6 cell death. To our knowledge, this is the first study to evaluate ART with 5-ALA-based SDT as a new technique for increasing SDT cytotoxicity. However, these results are only based on in vitro studies, and further in vivo studies are necessary.
